# A New Method of Plugging the Fracture to Enhance Oil Production for Fractured Oil Reservoir using Gel Particles and the HPAM/Cr^3+^ System

**DOI:** 10.3390/polym11030446

**Published:** 2019-03-08

**Authors:** Lei Zhang, Nasir Khan, Chunsheng Pu

**Affiliations:** 1 Department of Petroleum Engineering, China University of Geosciences (Wuhan), Wuhan 430074, China; zhangshishishi.188@163.com; 2 Department of Petroleum and Gas Engineering, Balochistan University of Information Technology, Engineering & Management Sciences (BUITEMS), Quetta 87300, Pakistan; nasir.khan1@buitms.edu.pk; 3 Institute of Petroleum Engineering, China University of Petroleum (East China), Qingdao 266580, China

**Keywords:** fracture, gel particle, HPAM/Cr^3+^ system, plugging, oil recovery

## Abstract

Due to the strong heterogeneity between the fracture and the matrix in fractured oil reservoirs, injected water is mainly moved forward along the fracture, which results in poor water flooding. Therefore, it is necessary to reduce the water cut and increase oil production by using the conformance control technology. So far, gel particles and partially hydrolyzed polyacrylamide (HPAM)/Cr^3+^ gel are the most common applications due to their better suitability and low price. However, either of the two alone can only reduce the conductivity of the fracture to a certain extent, which leads to a poor effect. Therefore, to efficiently plug the fracture to enhance oil recovery, a combination of gel particles and the HPAM/Cr^3+^ system is used by laboratory tests according to their respective advantages. The first step is that the gel particles can compactly and uniformly cover the entire fracture and then the fracture channel is transformed into the gel particles media. This process can enhance the oil recovery to 18.5%. The second step is that a suitable HPAM/Cr^3+^ system based on the permeability of the gel particles media is injected in the fractured core. Thus, the fracture can be completely plugged and the oil in the matrix of the fractured core can be displaced by water flooding. This process can enhance oil recovery to 10.5%. During the whole process, the oil recovery is increased to 29% by this method. The results show that this principle can provide a new method for the sustainable and efficient development of fractured oil reservoirs.

## 1. Introduction

Presently, there are a large number of fractured oil reservoirs, which play an important role in the oil industry in China [1,2,3,4]. During long-term water flooding, the fracture (including artificial fracture and natural fracture) is continuously washed, so that its size is further expanded [5,6]. Although the fracture can decrease the flow resistance of fluids and increase the initial production of the oil well, water can move forward along the fracture to the oil well, and increasing the water cut of the oil well dramatically. Therefore, a better choice to decrease the water cut is that the fracture is plugged using polymer gel and then the injected water can be diverted into the low permeability area [7].

During the operation of water plugging, one of the most common failure phenomena is that the polymer gel is easily penetrated by the subsequent water flow due to the defection of the polymer material. Thus, a new water channel is formed and the expansion of the swept volume is limited [8]. This is because plugging the fracture requires a polymer gel with a continuous body and a high mechanical strength, which can fully plug the water channel and maintain a good performance under the long-term washing of the injected water. Based on the field statistics of the Changqing oilfield and Yanchang oilfield, the most applied polymer gels are the gel particles and the in-situ cross-linking polymer gel. Gel particles have a dense three-dimensional network structure with a strong energy storage capacity, a high strength, and a high viscoelasticity [9,10]. However, due to the dispersed single bodies, it cannot form an integrity to fully fill in the fracture. In-situ cross-linking polymer gel consists of a high-molecular polymer and crosslinking agent, which is gelatinized with a continuity and integrity in the conditions of the reservoir [11]. However, due to a low concentration of the usually used polymer (1000–6000 mg/L), the formed gel is weak in mechanical strength. The injected water can easily break through the gel to form a new water channel [12]. Although increasing the concentration of the polymer can increase the strength of the gel, the initial viscosity of the gelant is also greatly increased, which can cause some problems, such as difficulties in stirring and pumping during on-site operation.

According to the characteristics of the dispersed single body of gel particles and the continuous bulk of the in-situ cross-linking polymer gel, the in-situ cross-linking polymer gel can consolidate the dispersed individual gel particles, while the gel particles can support the in-situ cross-linking polymer gel to increase the mechanical strength. Therefore, to further improve the utilization of gel particles and in-situ cross-linking polymer gel, the respective advantages of gel particles and in-situ cross-linking polymer gel are utilized together in this paper. The fracture is first transformed into porous media channels by using gel particles, and then the pore space of the gel particles media is plugged by the in-situ cross-linking polymer gel.

Regarding plugging the fracture through the use of gel particles, many scholars have done a lot of research due to its good adaptability, high utilization, and low price. In particular, Prof. Baojun Bai from Missouri University of Science and Technology has achieved many breakthrough achievements, which include the swelling and rheological behavior, plugging performance, areal sweep efficiency improvements by integrating PPG and low salinity water, propagation and dehydration, injection characteristics, effect of heterogeneous conduits and their geometrical on injectivity, effect of load pressure and back pressure on the gel pack permeability, optimization of the strength and size, etc. [9,13,14,15,16,17,18,19,20,21,22]. These results can give a clear understanding on the use of gel particles to plug fractures. However, after plugging using gel particles, the formed gel particles media in the fracture has a certain permeability and the low permeability layer cannot be swept. Therefore, it is necessary to further plug this channel to enhance oil recovery.

Regarding plugging the gel particles media, it is similar to plugging the porous media of a core by using an in-situ cross-linking polymer gel. Due to the strong adaptability and low price, in-situ polymer bulk gel has attracted more attention in conformance control, which has two components: High-molecular polymer and cross-linker. Under reservoir conditions, an in-situ three-dimensional network can thus be established, which displays a unique liquid-like behavior on a molecular length scale while maintaining solid-like macroscopic properties [23]. At the pore scale, two main shut-off mechanisms can be summarized [24,25,26,27,28]. One is in-depth flow diversion. In the heterogeneous layer, where water and oil exist in different channels, the injected water often cross-flows along high permeable water channels and bypasses low permeable pore throats. When the high permeability is plugged, the subsequent injected water is diverted to the adjacent low permeable pore throats, and thus the sweep efficiency is expanded. The other is water permeability reduction. As is well-known, after a period of water washing, a polymer gel may be broken through by the fluid. However, because of the high cementing between the gel and the rock, a gel film is formed on the wall of pore throats. Accordingly, the diameter of pore throats decreases and the permeability of the subsequent injected water is reduced. Based on these, the gel particles media can be further plugged to achieve the flow diversion.

Nonetheless, there are few reports on the combination of gel particles and in-situ crosslinking polymer gel to plug fractures. To this end, this paper uses an indoor experiment to combine the two operations to plug fractures and establish the plugging method and principles. The first step is that the size and injection parameters of the gel particles are optimized to plug the fracture. Then, a suitable in-situ crosslinking polymer gel is screened to plug the formed gel particles media and the gel particles can be locked up. Finally, the entire large fracture channel can be fully plugged to compel the subsequent water flow into the adjacent matrix porous media. This method can provide a new idea for the plugging of fractures in fractured oil reservoirs.

## 2. Experiment

### 2.1. Reagents and Equipment

In the experiment, the oil sample, with a viscosity of 7.1 mPa.s at the temperature of 25 ℃, was from Changqing oilfield, the largest oil-gas field in China. Simulated formation water was prepared with 10,000 mg/L NaCl and deionized water, which was used to prepare all the solutions. Gel particles with a crosslinking density of 14% were obtained from Shandong Shida Oilfield Technical Services Co., Ltd. in Shandong, China, which were formed by the copolymerization of a certain proportion of modified starch, acrylamide, N’N-methylene bis acrylamide, and initiator after being dried and smashed. A series of the particle sizes of the gel particles were screened out by the sieves with the different pore sizes. The HPAM/Cr^3+^ system consisted of partially hydrolyzed polyacrylamide (HPAM) and chromium acetate, which has been studied extensively and reported in detail [29,30]. HPAM with a molecular weight of 12 million g/mol and a hydrolysis degrees of 25% was obtained from Beijing Hengju Chemical Co., Ltd. in Beijing, China. Chromium acetate solution was also obtained from Shandong Shida Oilfield Technical Services Co., Ltd. The concentration of Cr^3+^ was 5 mg/mL.

A series of artificial cores were bought from Research Institute of Exploration and Development of CNPC, which were made by the cementation of quartz sand and epoxy resin. The parameters of the cores are shown in Table 1. Other instruments included a Haake RS600 rheometer (Thermo Scientific, Waltham, MA, USA), a DV-III Brookfield viscometer (Brookfield Company, Middleboro, MA, USA), and a GE ProSpeed single-slice spiral CT (GE Healthcare, Pittsburgh, PA, UAS). The viscosities of each of the samples were measured at a shear rate of 7.34 s^−1^ by the viscometer and the elastic modulus of the gels were measured at the different shear rates by the rheometer at room temperature.

### 2.2. Fracture Model

**Figure 1:**
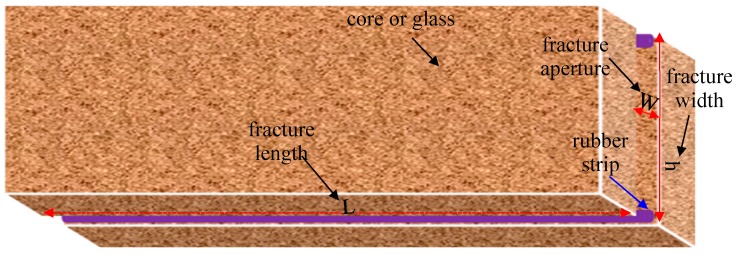
Fracture models (two thin rubber strips between two cores or two glass).

**Figure 2:**
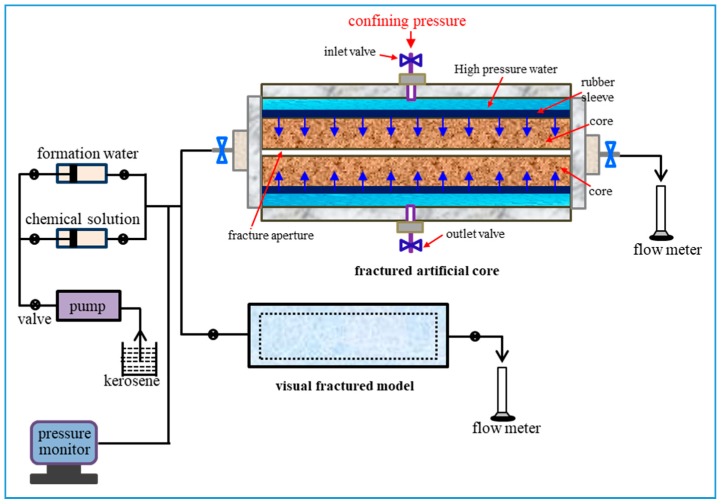
Simulation experiment device for displacement.

The visual fractured model with a length of 30 cm and a width of 4.5 cm was made by using epoxy resin adhesive to cement two pieces of single frosted glass with two thin rubber strips between them along the sides. During displacement, the image was obtained using a CCD camera at any time. The fractured artificial core was made by fracturing an artificial core with a water permeability of 10 × 10^−3^ μm^2^ along the injection direction from the middle part, and then two thin rubber strips were filled in the fractures along the two sides. The two thin rubber strips had the same size with a thickness of 2 mm. The fractured artificial core model is shown in Figure 1. The total pore volume of a whole fractured core (TPV) consists of the volume of the fracture (FPV) and the pore volume of the matrix (MPV). 1 MPV is 90 cm^3^, 1 FPV is 30 cm^3^, and 1 TPV is 120 cm^3^. The simulation experiment device for displacement is shown in Figure 2. A special core gripper was applied for continuous and uninterrupted computed tomography (CT) scanning, which was obtained from Jiangsu Huaan Scientific Research Devices Co., Ltd in Jiangsu, China. In the experiment, the confining pressure was fixed at 5 MPa to seal the fractured core. Due to the weak compressible, the change of the thickness of rubber under the pressure of 5 MPa was ignored. Besides, as the outlet is directly connected to the atmosphere, the injection pressure of a fluid is equal to its flow resistance across the fractured model.

### 2.3. Experimental Procedure

The basic principle of the experiment is that the fracture is first filled with the gel particles to form a new porous medium, and then the formed gel particles medium is plugged by an HPAM/Cr^3+^ system. During this process, the injection parameters of the gel particles and the HPAM/Cr^3+^ system are crucial for the transportation and plugging. Therefore, optimization of the injection parameters can maximize the plugging effect.

#### 2.3.1. Optimization of the Gel Particle Size

After screening and water swelling in the simulated formation water, the sizes of the gel particles with a density of 1.09 g/cm^3^ were 2.2 mm, 2.6 mm, 3 mm, 3.6 mm, and 4.5 mm, respectively. Next, each type of gel particles solutions with a concentration of 5000 mg/L was prepared with the simulated formation water. Thus, each was injected into the fractured core at an injection rate of 2 mL/min at room temperature. Changes in the injection pressures were recorded during the displacement until the pressure reaches an equilibrium state. Finally, the matching relationship of the size of the gel particles and the fracture aperture was established.

#### 2.3.2. Optimization of the Injection Parameters of Gel Particles

It is well known that reasonable injection parameters can allow more gel particles to enter the fracture with a uniform distribution, which can increase the plugging strength and expand the swept volume to a greater extent [17,18,19]. The injection parameters mainly include the concentration and the injection rate of gel particles solutions, which can be collectively reflected by the injection mass of the gel particles per unit of time. Therefore, it is reasonable to keep the mass flow rate constant and to adjust the injection concentration and the injection rate of gel particles during the compilation of an injection parameters program.

Based on the results in Section 2.3.1, a suitable particle size was screened for the optimization of the injection parameters. The mass flow rate of the gel particles was fixed at 10 mg/min according to feedback from the on-site construction of gel particles from the Shengli oilfield in Dongying, China. Thus, six schemes were designed as the test subjects, which are as follows: (1) Injection concentration of 500 mg/L and injection rate of 20 mL/min; (2) injection concentration of 1000 mg/L and injection rate of 10 mL/min; (3) injection concentration of 2000 mg/L and injection rate of 5 mL/min; (4) injection concentration of 5000 mg/L and injection rate of 2 mL/min; (5) injection concentration of 10,000 mg/L and injection rate of 1 mL/min; (6) injection concentration of 20,000 mg/L and injection rate of 0.5 mL/min. In the experiment, six visual fracture models were used for the six schemes to observe the characteristics of the distribution and the leading displacement front of the gel particles during transportation at room temperature. During the implementation of a scheme, when the gel particles reached the outlet or the pressure was increased sharply, the experiment was stopped and then the next scheme was executed until the six schemes were completed.

#### 2.3.3. Testing the Threshold Pressure of Transportation in the Fracture of the Gel Particles

After a fracture is compactly filled with gel particles, it is transformed into a gel particles media, which can be taken as an unconsolidated porous media. Due to the frictional resistance and wall surface extrusion on the gel particles, the unconsolidated gel particles can be pushed only when the displacing pressure of a fluid is greater than the threshold pressure of transportation in the fracture (P_T_) of the gel particles. Thus, when the injection pressure is higher than the P_T_, the distribution of the gel particles in the fracture and the permeability of the gel particles media are changed, which may lead to a wrong guide for the selection of the HPAM/Cr^3+^ system. Therefore, the injection pressure of the subsequent fluid needs to be lower than the P_T_ of the gel particles.

When the gel particles are displaced, the permeability of the gel particles media is changed, which can cause the linear relationship between the flow rate and difference pressure to change based on Darcy’s formula. Consequently, the P_T_ of the gel particles can be characterized by the mutation of the linear relationship between the flow rate and difference pressure. The specific experimental method is as follows: (1) A gel particles solution with the appropriate parameters based on the experimental results in Section of 2.2.2 was injected into a fractured core until the gel particles reached the outlet and the injection pressure became stable; (2) the pressure drop of the subsequent water flooding across the fractured core was measured at different injection rates in the range of 0.2 mL/min to 1.2 mL/min from low to high. Thus, the changes in the injection pressures with the injection rates were recorded.

The calculation formula of permeability is as follows:

(1) $$k_{total}=\frac{Q\mu L}{A\Delta P}$$

(2) $$k_{gel}=k_{total}-k_{\emptyset }$$

For the fractured core, $k_{\emptyset }$ is 10 × 10^−3^μm^2^; *A* is 20.5 cm^3^; and *L* is 30 cm.

#### 2.3.4. Optimization of Parameters of the HPAM/Cr^3+^ System for Plugging the Gel Particles Media

For plugging the gel particles media, an artificial core with a same/higher water permeability to/than that of the gel particles media was used in the experiment. This is because it is only when the plugging strength of a HPAM/Cr^3+^ gel in the artificial core is higher than the flow resistance of water in the matrix of the fractured core that the gel particles media can be completely plugged to divert the followed fluids into the matrix. The plugging strength is characterized by measuring the breakthrough pressure of the subsequent water flow across the artificial core after it is plugged by the HPAM/Cr^3+^ gel. The flow resistance of water in the matrix of the fractured core is equivalent to that of water in an artificial core with a water permeability of 10 × 10^−3^ μm^2^, of which the flow resistance can be measured. Thus, the compositions of the HPAM/Cr^3+^ system can be screened based on the required plugging strength of the gel.

**Figure 3:**
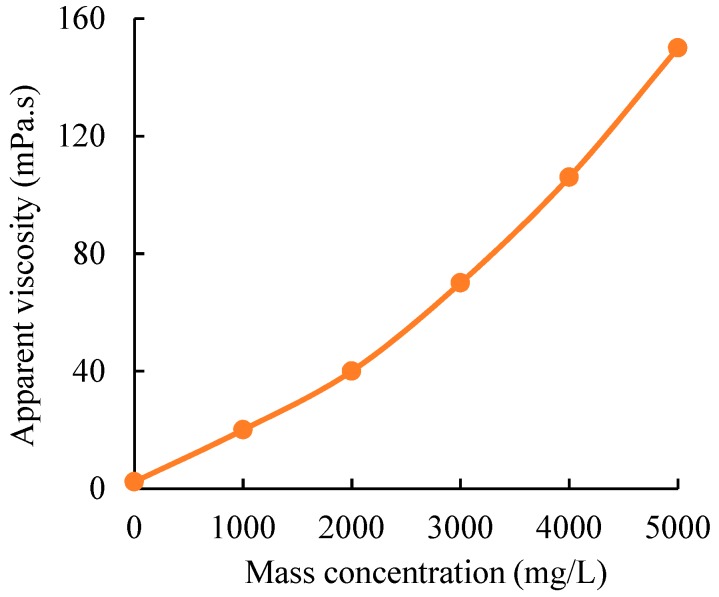
Corresponding values of the viscosity of the partially hydrolyzed polyacrylamide (HPAM) solution versus the mass concentration of HPAM.

However, it should be noted that to ensure that the gel particles do not migrate, the injection pressure of the HPAM/Cr^3+^ system is less than the P_T_ of the gel particles. Therefore, the injection rate of the HPAM/Cr^3+^ system is limited when the composition of the HPAM/Cr^3+^ system is fixed. At the initial stage, the viscosity of the HPAM/Cr^3+^ system is equal to that of the HPAM solution with the same concentration. The corresponding values between the viscosity of the HPAM solution and its mass concentration are shown in Figure 3. Therefore, the calculation formula of the limited injection rate of the HPAM/Cr^3+^ system is as follows:

(3) $$\Delta P_{HPAM}\leq P_{T}$$

(4) $$\frac{Q_{HPAM}\mu_{HPAM}L}{Ak_{gel}}\leq P_{T}$$

(5) $$Q_{HPAM}\leq \frac{P_{T}Ak_{gel}}{L\mu_{HPAM}}$$

Similarly, for the fractured core, L is 30 cm and *A* is 20.5 cm^3^.

When an appropriate HPAM/Cr^3+^ system is injected into the gel particles media, not only do the gel particles become locked up, but also their pore space can be plugged after gelation.

#### 2.3.5. Testing the Plugging Effect of the HPAM/Cr^3+^ System Assisting Gel Particles in the Fractured Core

After plugging the fracture water channel, it is necessary to analyze the effect of enlarging the swept volume of water flooding into the heterogeneous oil reservoir. For sandstone and carbonate reservoirs, CT scanning can be used to display the main parameters (e.g., density distribution and oil–water saturation distribution) in the porous media and fracture media quantitatively and quickly without destroying the media, thus it is an accurate and clear method to evaluate the increment of oil recovery before and after plugging. In our previous studies [31], the basic principle, methods, and parameters of the application of CT technology in conformance control of heterogeneous porous media was reported in detail. Based on this, CT technology was used to observe the variation of the swept volume before and after plugging the large fracture.

In the experiment, an artificial core with a water permeability of 1000 × 10^−3^ μm^2^ was used to be processed into a fractured core. Thus, the fractured core was used in the flow experiment. The detailed experimental procedure is as follows: (1) The core with a water permeability of 1000 × 10^−3^ μm^2^ was first vacuumized and then saturated with water; (2) the core with a water permeability of 1000 × 10^−3^ μm^2^ was flooded with the oil sample until the effluent was all oil; (3) the core with a water permeability of 1000 × 10^−3^ μm^2^ was processed into a fractured core based on the above method; (4) the fractured core was displaced by the simulated water at an injection rate of 0.5 mL/min until the flow rates at the inlet and the outlet were the same and the injection pressure began to stabilize. After, the fractured core was scanned; (5) according to the optimization results in Section 2.3.1 and Section 2.3.2, a certain volume of gel particles solution was injected; (6) the subsequent water flooding was carried out on the condition that the injection pressure was lower than the P_T_ of the gel particles until the water cut of the effluent was 98%, and then the fractured core was scanned; (7) after, the pore volume of the gel particles media (GPV) was calculated according to the difference between the volume of the fracture (FPV) and that of the gel particles (injection mass divided by density), 1 GPV of the HPAM/Cr^3+^ system was injected into the fractured core; (8) after gelation of the HPAM/Cr^3+^ system, the subsequent water flooding was carried out again until the water cut of the effluent was 98%, and then the fractured core was scanned. Thus, changes in the swept volume before and after plugging were obtained.

## 3. Results and Discussion

### 3.1. Matching Relationship of the Size of Gel Particles and Fracture Aperture and its Mechanical Mechanism

**Figure 4:**
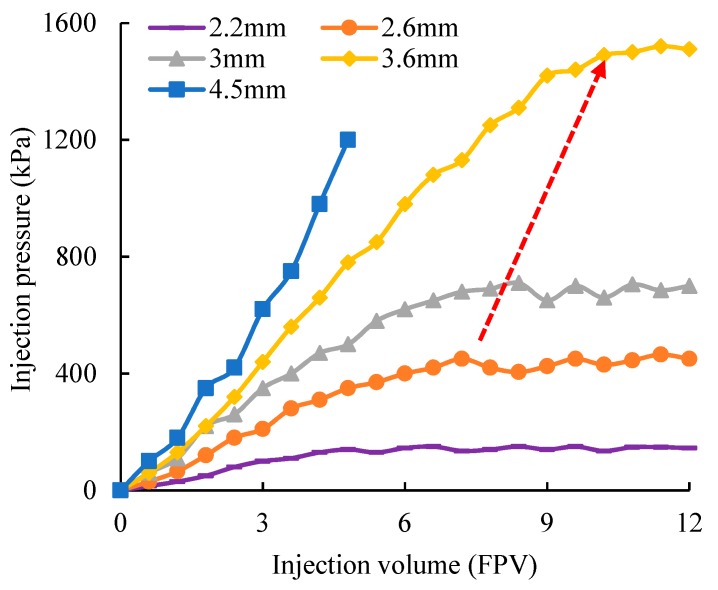
Relationship between the injection pressure and the particle size.

The flow resistance of gel particles with different particle sizes in the fractured core is shown in Figure 4. When the particle size is low, the gel particles can smoothly pass through the fracture and cannot plug, such as the curve of the gel particles with a particle size of 2.2 mm. However, when the particle size is too large, the flow resistance sharply increases. This is because the size of the gel particles after deformation is still larger than the fracture aperture. Thus, the accumulation of gel particles at the inlet occurs and they cannot enter into the depth of the fracture, such as the curve of the gel particles with a particle size of 4.5 mm. Only when the particle size is appropriate, can the gel particles enter into the fracture with a better flow resistance, such as the curve of the gel particles with a particle size in the range of 2.6–3.6 mm. Moreover, within this range, with the increase of the particle size, the balance point of the injection pressure on the curve moves to the right and the amount of injection is also increased, which shows that the larger the particle size, the greater the amount of particles remaining in the fracture. The experimental results show that the larger the particle size, the better the plugging is under the premise that the gel particles can smoothly enter into the fracture.

### 3.2. Effect of Injection Parameters on the Distribution and Flow of Gel Particles in the Fracture

**Figure 5:**
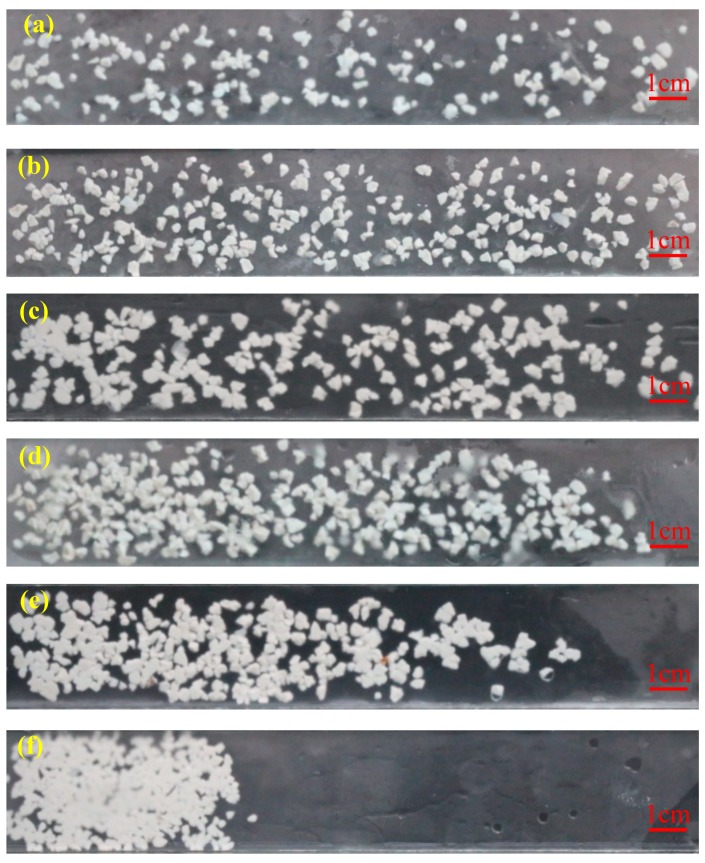
Distribution of the gel particles in the fracture in the condition with different injection schemes ((**a**): injection concentration of 500 mg/L and injection rate of 20 mL/min; (**b**): injection concentration of 1000 mg/L and injection rate of 10 mL/min; (**c**): injection concentration of 2000 mg/L and injection rate of 5 mL/min; (**d**): injection concentration of 5000 mg/L and injection rate of 2 mL/min; (**e**): injection concentration of 10,000 mg/L and injection rate of 1 mL/min; (**f**): injection concentration of 20,000 mg/L and injection rate of 0.5 mL/min).

**Figure 6:**
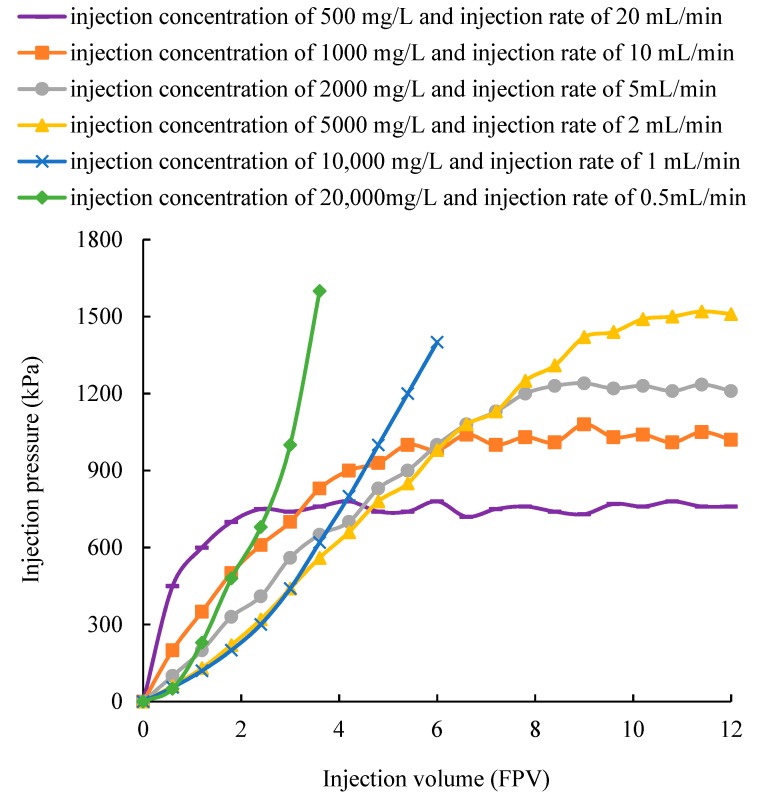
Changes in the injection pressure under the different injection parameters.

According to the results in Section 3.1, the gel particles with a size of 3.6 mm were selected and then injected into the fracture. Its distribution and flow resistance in the fracture under the same mass flow rate and different injection schemes are shown in Figure 5 and Figure 6, respectively. Figure 5a shows that a low concentration and a high flow rate can produce a high flushing action on the gel particles, so that the gel particles can smoothly pass through the fracture and its distribution in the fracture is scattered. In addition, due to a high flow rate, the injection pressure is rapidly increased at the early stage, but is kept at a low level at the later stable stage, such as the curve under an injection concentration of 500 mg/L and an injection rate of 20 mL/min in Figure 6. This can lead to a poor plugging effect. When the concentration and flow rate are both moderate, the gel particles can densely cover the whole fracture, which is shown in Figure 5b–d. Correspondingly, the injection pressure is gradually and steadily increased and can maintain a high value at the stable stage, such as the curve under an injection concentration of 1000 mg/L and an injection rate of 10 mL/min, an injection concentration of 2000 mg/L and an injection rate of 5 mL/min, and an injection concentration of 5000 mg/L and an injection rate of 2 mL/min in Figure 6. By further comparison, we can conclude that the injection volume of gel particles is the largest and the injection pressure is the highest under an injection concentration of 5000 mg/L and an injection rate of 2 mL/min, which is advantageous for plugging the whole fracture. As the concentration is further increased and the injection rate is further reduced, the distribution of gel particles in the fracture becomes more compact and tight until the accumulation occurs at the inlet and causes a sharp increase in the pressure, as shown in Figure 5e,f, and the curve under an injection concentration of 10,000 mg/L and an injection rate of 1 mL/min, and an injection concentration of 20,000 mg/L and an injection rate of 0.5 mL/min in Figure 6. After comprehensive analysis, it is concluded that an injection concentration of 5000 mg/L and an injection rate of 2 mL/min is optimal for plugging.

### 3.3. Characteristics of the Leading Displacement Front of the Gel Particles During Transportation

**Figure 7:**
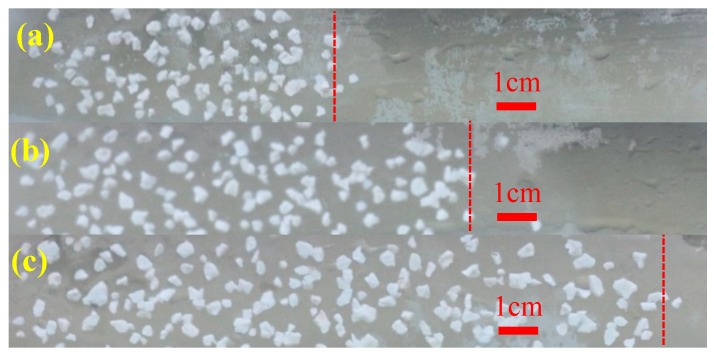
Characteristics of the leading displacement front of the gel particles in the fracture with different injection volumes ((**a**): injection of 5 FPV; (**b**): injection of 7 FPV; (**c**): injection of 10 FPV).

According to the results in Section 3.1 and Section 3.2, the gel particles with a size of 3.6 mm were injected into the fracture under the concentration of 5000 mg/L and an injection rate of 2 mL/min (this set of parameters was used in the following experiment). The observed characteristics of the leading displacement front of the gel particles during transportation are shown in Figure 7, which shows that the leading displacement front advances more uniformly in a piston type. Based on this characteristic, the distance of transportation of the gel particles in the fracture can be obtained by combining the distribution and the injection volume of the gel particles in the fracture. In addition, the relationship between the injection volume of the gel particles and the filled area of the fracture can also be quantitatively characterized. According to the comprehensive analysis of Figure 5, Figure 6 and Figure 7, the whole fracture can be compactly filled by gel particles when the injecting volume is 10 FPV.

The gel particles are distributed in a single form in the fracture, which means that the transportation of the gel particles in the fracture is the movement an of individual gel particle. The force characteristics of the whole gel particles can be explained by that of a single gel particle.

**Figure 8:**
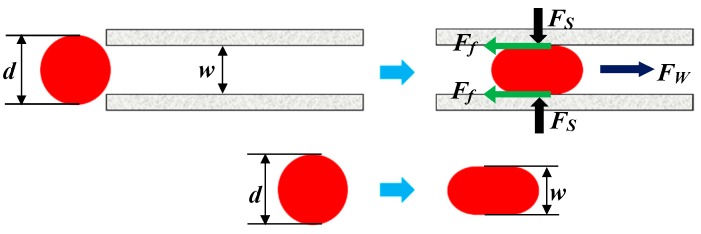
Diagram of the transportation in the fracture of a gel particle.

When a gel particle is transported and deformed in the fracture under the action of water flow and the wall of the fracture, its shape can be taken as a spherical shape under ideal conditions, which is shown in Figure 8. When it is compressed, the particle contacts with the wall surface of the fracture by surface contact rather than point contact. Therefore, the gel particle is subjected to three forces, which are the pressure of the wall surface of the fracture on the gel particles (*F_S_*), the frictional resistance of the gel particle moving forward (*F_f_*), and the water flow flushing force (*F_w_*), respectively [32].

It can be assumed that the deformation of a gel particle during transportation in the fracture belongs to the type of elastic deformation. Next, the additional flow resistance generated when the particle is squeezed in the fracture can be caused by the comprehensive effect of elastic deformation and the internal stress of the particle. Thus, according to the principle of elastic mechanics and the principle of balance, a mathematical expression can be obtained, which is as follows:

(6) $$\Delta P=\sigma_{1}=\sigma_{p}$$

According to the general Hooke’s law, the expression of elastic strain of a gel particle after being compressed in the fracture can be obtained, which is as follows:

(7) $$\varepsilon =\frac{1}{E}\left(\sigma_{p}-\nu \sigma_{1}\right)$$

Next, according to Equations (6) and (7), the flow resistance in the fracture of gel particles can be obtained, which is as follows:

(8) $$\Delta P=\frac{E}{1-\nu }\varepsilon$$

Thus, the strain of the gel particles in the fracture can be calculated according to the size of the gel particles and the aperture of the fracture, which is as follows:

(9) $$\varepsilon =1-\frac{w}{d}$$

Finally, when Equation (9) is substituted into Equation (8) and a frictional resistance coefficient is also introduced into Equation (8), a new Equation can be obtained, which is as follows:

(10) $$\Delta P_{f}=f\cdot \frac{E}{1-\nu }\cdot \left(1-\frac{w}{d}\right)$$

According to Equation (10), when the size of the gel particles and the aperture of the fracture are fixed, the flow resistance is proportional to the friction coefficient. It is known that the friction coefficient is proportional to the injection volume of the gel particles in the crack, so that the flow resistance is proportional to the injection volume (e.g., the characteristics of the curve of scheme 4 shown in Figure 5) and the gel particles move forward in a piston fashion.

### 3.4. Threshold Pressure of the Gel Particles During Transportation

**Figure 9:**
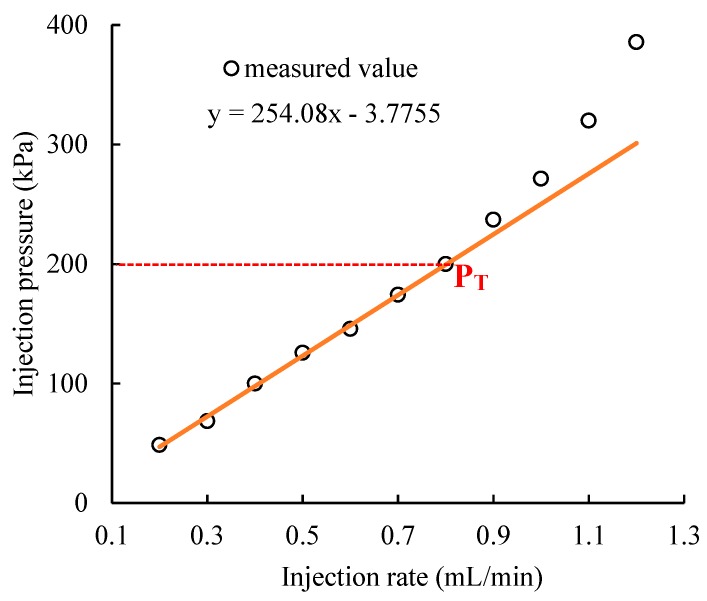
Changes in the injection pressure with the injection rate.

The changes in injection pressure with the injection rate are shown in Figure 9, which show that the linear relationship between the injection pressure and injection rate is changed when the injection pressure is more than 200 kPa and the injection rate is more than 0.8 mL/min. Beyond this range, gel particles can be pushed. Therefore, the P_T_ is 200 kPa and the permeability of the gel particles media is 8050 × 10^−3^ μm^2^ in the linear region according to Darcy’s formula.

### 3.5. Composition of the HPAM/Cr^3+^ System for Plugging the Gel Particles Media

**Figure 10:**
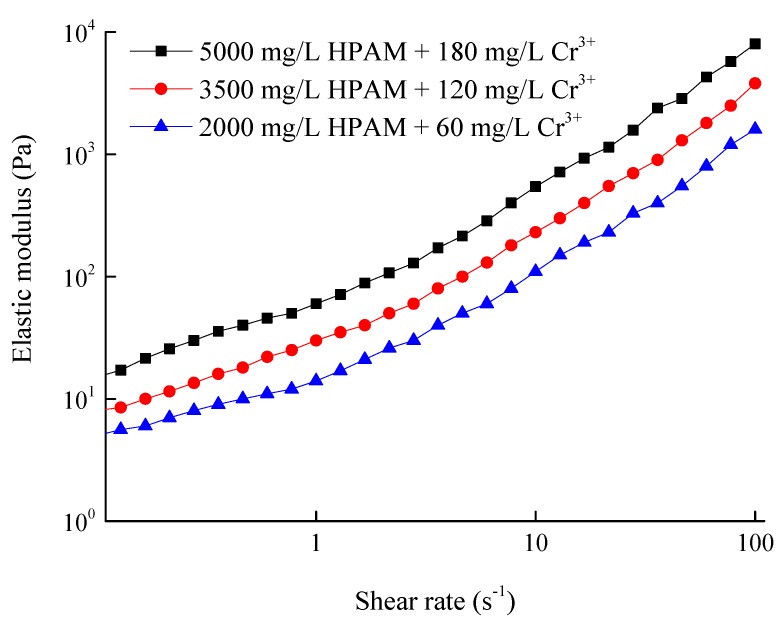
Relationship of the elastic modulus and the shear rate of the three gels.

According to the experimental results in Section 3.4, the artificial core with a water permeability of 9000 × 10^−3^ μm^2^ was taken as a testing object, of which the permeability was higher than that of the gel particles media (8050 × 10^−3^ μm^2^). After vacuumization and saturation with water, the HPAM/Cr^3+^ systems with the different parameters were respectively injected into the core, and the experimental results are shown in Table 2. On the other hand, the gradient of the water flow resistance in the core with a water permeability of 1000 × 10^−3^ μm^2^ was 50 kPa/m at a flow rate of 1 mL/min. When the gradient of the breakthrough pressure of the water flowing through the core with a water permeability of 9000 × 10^−3^ μm^2^ after being plugged using the HPAM gel was higher than 50 kPa/m at a flow rate of 1 mL/min, the water in the matrix of the fractured core was displaced, which is equivalent to the flow resistance in the core with a water permeability of 1000 × 10^−3^ μm^2^. According to the results in Figure 10 and Table 2, for gel particles media with a permeability of 9000 × 10^−3^ μm^2^, after being plugged by the HPAM gel with a composition of 3500 mg/L HPAM and 120 mg/L Cr^3+^, the pore space of the gel particles media can be completely plugged to divert the flow into the matrix. In addition, according to Formula (5), the injection rate of the HPAM/Cr^3+^ system (3500 mg/L HPAM + 120 mg/L Cr^3+^) with an initial viscosity of 80 mPa.s must be lower than 0.1 mL/min to ensure that the flow resistance of the HPAM/Cr^3+^ system in the gel particles media is below 200 kPa. Thus, the HPAM/Cr^3+^ system with 3500 mg/L HPAM and 120 mg/L Cr^3+^ can be applied to plug the fracture core.

### 3.6. Changes in the Swept Volume by Water Flooding Before and After Plugging

**Figure 11:**
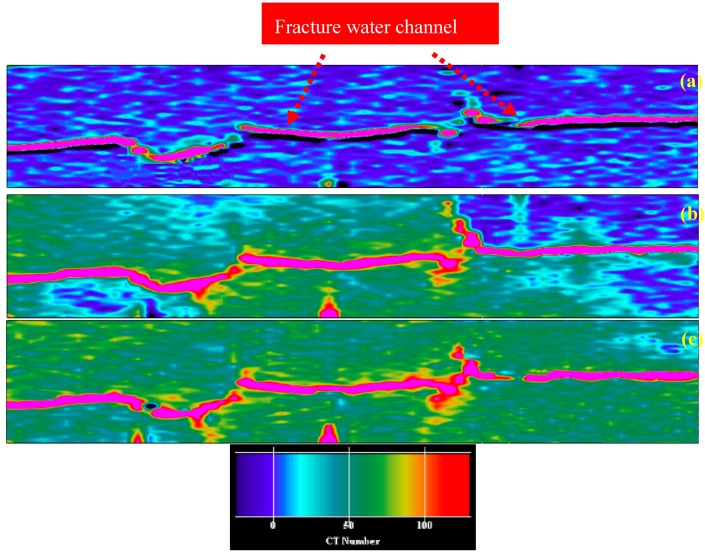
Profile of fluids distribution in the fractured core by water flooding before and after plugging ((**a**): before plugging; (**b**): after plugging using gel particles; (**c**): after plugging using HPAM/Cr^3+^ system. Besides, the flow direction is from left to right).

Based on the above results, 10 FPV of the gel particles solution with a concentration of 5000 mg/L was first injected into the fractured core at an injection rate of 2 mL/min, and then water flooding was carried out until the water cut was increased to 98%. Secondly, 12 cm^3^ (1GPV) of the HPAM/Cr^3+^ system (3500 mg/L HPAM + 120 mg/L Cr^3+^) was subsequently injected into the fractured core at an injection rate of 0.04 mL/min. After gelation of the HPAM/Cr^3+^ system, water flooding was carried out again until the water cut was increased to 98%. Thus, the characteristics of the changes in the swept volume by water flooding before and after plugging using polymer gel assistance to the gel particles was obtained through CT scanning, which are shown in Figure 11. The blue region is the oil, the red region is the fracture channel, and the green region is water. Figure 11a shows that the injected water basically flows in the fracture channel and the oil in the matrix was not swept and displaced. Figure 11b shows that the fracture was plugged by the gel particles, and the swept volume increased to a certain extent. However, there are still some areas that were swept. This shows that the heterogeneity of the fractured core plugged by gel particles needs to be further improved. Thus, the HPAM/Cr^3+^ system was injected into the gel particles and the blue region was further reduced and the green region was further increased, which is shown in Figure 11c. We can conclude that when a suitable HPAM/Cr^3+^ system is applied based on the optimization of the injection parameters of gel particles, the oil in the matrix can be almost completely swept during water flooding.

**Figure 12:**
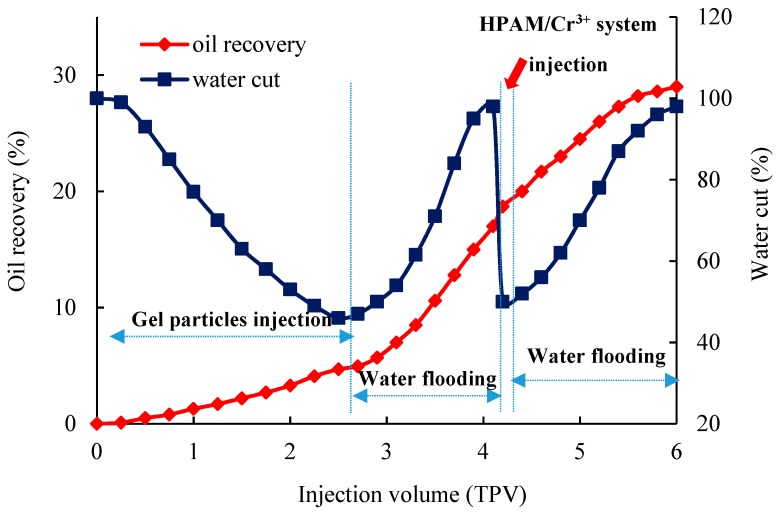
Characteristics of the oil recovery in the fractured core during the gel particles injection, water flooding, HPAM/Cr^3+^ system injection, and subsequent water flooding.

The change of the oil recovery during plugging using gel particles and the HPAM/Cr^3+^ system in the fractured core is shown in Figure 12. Owing to the strong heterogeneity of the fracture, the water cut of the primary water flooding was almost 100%. With the increases of the injection of the gel particles solution, the fracture was gradually plugged and the oil in the matrix was gradually displaced by the water flow from the fracture to the matrix. When the fracture was completely filled with gel particles, the water cut reached a minimum. Next, water flooding was carried out and the water cut was gradually increased to 98%, and the oil recovery was significantly enhanced to 18.5%. Thus, the HPAM/Cr3+ system was injected in the fractured core. After gelation, water flooding was carried out again and the water cut was gradually increased to 98% again, and the oil recovery increased by 10.5%. The whole process enhanced the oil recovery to 29%.

## 4. Conclusion

Based on the respective advantages, the gel particles and HPAM/Cr^3+^ system were combined be for the plugging of fracture water channels to enhance the oil recovery of fractured oil reservoirs.

(1) The HPAM/Cr^3+^ system can consolidate the dispersed individual gel particles; conversely, the gel particles with a high strength can support the HPAM/Cr^3+^ gel. The two together can improve the plugging strength in the fracture.

(2) Within a certain range, the larger the particle size, the greater the amount of gel particles retained in the fracture. The larger the particle size, the better the plugging effect.

(3) When the concentration and flow rate are suitable, the gel particles can densely and uniformly cover the whole fracture, which is beneficial for plugging. Besides, the leading displacement front of the gel particles in the fracture advances more uniformly in a piston type, and the depth of transportation can be obtained by combining the distribution in the fracture and the amount of the injection of the gel particles.

(4) According to the permeability of the gel particles media and the threshold pressure of transportation in the fracture of the gel particles, a suitable HPAM/Cr^3+^ system can be used to plug the pore channel of the gel particles media, which can plug the whole large-scale fracture and the subsequent water flooding can be diverted into the matrix to displace the oil.

(5) After being plugged by the gel particles, the oil recovery can be increased by 18.5%. Thus, after being plugged by HPAM/Cr^3+^ system, the oil recovery can be increased by 10.5%. By the proposed method, oil recovery was increased to 29%.

## Figures and Tables

**Table 1:** Parameters of the artificial cores used in the flow experiment.

No.	Number of cores	Water permeability (10^−3^μm^2^)	Porosity (%)	Size		
				Length (cm)	Width (cm)	Height (cm)
1#	6	1000	18.2	30	4.5	4.5
2#	5	9000	22.5	30	4.5	4.5

**Table 2:** Parameters of the experiment for testing the breakthrough pressure.

No.	Permeability of the core (10^−3^μm^2^)	Composition of HPAM/Cr^3+^ system	Initial viscosity of HPAM/Cr^3+^ system (mPa.s)	Gelation time (h)	Elastic modulus of the HPAM/Cr^3+^ gel at shear rate of 1s^−1^	Gradient of breakthrough pressure at a water flow rate of 1 mL/min (kPa/m)
1#	9000	5000 mg/L HPAM + 180 mg/L Cr^3+^	150	33	60	130
2#		3500 mg/L HPAM + 120 mg/L Cr^3+^	80	42	30	85
3#		2000 mg/L HPAM + 60 mg/L Cr^3+^	40	50	12	35

## Nomenclature

*W* = aperture of the fracture;
*h* = width of the fracture;
*d* = diameter of the gel particle;
*F_S_* = forced pressure of the wall surface of the fracture on the gel particles;
*F_f_* = frictional resistance of the gel particle moving forward;
*F_w_* = water flow flushing force;
Δ*P* = flow resistance in the fracture of a fluid, kPa;
*σ_1_* = elastic energy storage of gel particles, kPa;
*σ_P_* = internal stress of gel particles, kPa;
*ε* = strain of a gel particle, dimensionless;
*E* = elastic modulus of the gel particle, kPa;
*v* = Poisson’s ratio of the gel particle, dimensionless;
Δ*P_f_* = flow resistance of a gel particle, kPa;
*f* = frictional resistance coefficient, dimensionless;
*k_total_* = water permeability of the fractured core after plugged by gel particles, 10^−3^μm^2^;
*k_gel_* = water permeability of gel particles, 10^−3^μm^2^;
*k_Φ_* = water permeability of the matrix of the fractured core, 10^−3^μm^2^;
*Q* = injection rate, mL/min;
*S* = cross-sectional area of the fractured core, cm^2^;
*L* = length of the fractured core, cm;
*μ* = viscosity of a fluid, mPa.s.
Δ*P_HPAM_* = injection pressure of HPAM/Cr^3+^ system, kPa;
*μ_HPAM_* = viscosity of HPAM solution, mPa.s.
*Q_HPAM_* = injection rate of HPAM solution, mL/min.
*P_T_* = threshold pressure of transporting in the fracture of the gel particles, kPa.
